# Imaging-detected sports injuries and imaging-guided interventions in athletes during the 2022 FIFA football (soccer) World Cup

**DOI:** 10.1007/s00256-023-04451-z

**Published:** 2023-09-16

**Authors:** Marcelo Bordalo, Andreas Serner, Eduardo Yamashiro, Emad Al-Musa, Mohamed Abdelatif Djadoun, Khalid Al-Khelaifi, Yorck Olaf Schumacher, Abdulaziz Jaham Al-Kuwari, Andrew Massey, Pieter D’Hooghe, Marco Cardinale

**Affiliations:** 1 Aspetar Orthopedic and Sports Medicine Hospital, Doha, Qatar; 2 Fédération Internationale de Football Association (FIFA), Zurich, Switzerland

**Keywords:** MRI, US, Radiography, Musculoskeletal, Sports injuries, Radiology, Image-guided procedure, Soccer, Football

## Abstract

**Objective:**

To describe imaging-detected musculoskeletal injuries and image-guided interventional procedures during the 2022 FIFA football (soccer) World Cup.

**Materials and methods:**

Retrospective analysis of all radiologic examinations performed in a central medical facility for athletes was performed by two board certified musculoskeletal radiologists. Data on muscle, tendon, ligament, cartilage, and bone injuries were collected according to imaging modality and body part.

**Results:**

A total of 143 radiology examinations in 94 athletes were evaluated at the central medical facility. Magnetic resonance imaging (MRI) was the most utilized modality (67%), followed by radiography (12%), ultrasonography (9%), and computed tomography (4%). Image-guided interventions corresponded to 8% of all radiological examinations. There were 112 injuries described, affecting muscles and tendons (42%), ligaments (25%), cartilage (21%), and bone (12%). Most injured body parts were thigh (27%), foot and ankle (23%), knee (23%), and hip/groin (8%). Most injured players were within the age range of 24–35 years old (71%).

**Conclusion:**

Imaging was utilized in 11% of players who participated in the 2022 FIFA World Cup in Qatar. MRI was the most utilized modality, and acute muscle tears were the most diagnosed type of injury.

**Summary:**

Diagnostic imaging played an important role in diagnosing sports-related injuries during the 2022 FIFA World Cup.

## Introduction

The FIFA football (soccer) World Cup is the biggest sport event in the world along with the Olympic Games. It is normally held in multiple cities with large distances between stadiums. The 2022 World Cup was held in Qatar with, overall, 832 athletes from 32 nations participating in the competition. All the stadiums were located within a 55-km radius of Qatar’s capital city of Doha, which allowed, for the first time in a FIFA World Cup, a centralized organization of athlete medical health and performance care.

Injuries are a challenge for players and medical staff during major championships due to the high stakes and short duration between matches. Injury surveillance at the FIFA World Cups has been performed since 1998 providing an overview of typical injuries observed during matches [1, 2]. The use of imaging services throughout the tournament has not been reported. In comparison, research from the Olympic Games demonstrates a high reliance on radiology assessments as one of the main medical services required [3–5].

Radiology support in major tournaments is not only provided for diagnostic purposes but also for specific medical interventions. An overview of imaging services required during a FIFA World Cup can provide a deeper insight into injuries occurring at the highest level and help optimize medical care in future events.

To the best of our knowledge, no epidemiological data regarding imaging-detected musculoskeletal injuries or imaging-guided interventions in a major football event have been published. Qatar 2022 was the first FIFA World Cup in which medical services to the players were performed in a central facility, thus enabling a unique overview of imaging-detected injuries among athletes during competition.

We aim to describe epidemiological data regarding imaging-detected musculoskeletal injuries and imaging-guided interventional procedures among players during the 2022 FIFA World Cup who attended the central medical facility (Aspetar Orthopedic and Sports Medicine Hospital).

## Materials and methods

We performed a retrospective cohort study approved by the institutional review board (IRB number E202211048). Informed consent was waived because all data were anonymized.

Radiology services were available at all hours from November 10 (10 days before the first match of the tournament) to December 19 (1 day after the World Cup final). Images were taken using one digital radiography (XR) machine (Digital Diagnost, c90, Philips Healthcare, Netherlands), two ultrasound (US) machines (Aplio i800, Canon Medical, Otawara, Japan and Acuson Juniper, Siemens Healthineers, Erlangen, Germany), one 64-slice computed tomography (CT) scanner (Somatom Definition AS, Siemens Healthineers, Erlangen, Germany), and two magnetic resonance (MR) imaging magnets (3.0 T Discovery 750 w, GE Healthcare, Milwaukee, USA and 1.5 T Magnetom Altea, Siemens Healthineers, Erlangen, Germany).

All referrals were made by a team physician. In each instance, the clinical indication was related to a recent injury sustained during a match or training, either accompanied by pain and physical limitations or included as a follow-up of a recent injury (within a maximum, 3-month window). The choice of imaging modality was determined by the team physician’s discretion and, in some instances, by mutual agreement with the staff radiologist.

Imaging data were retrospectively collected through the Picture Archiving and Communication System (PACS) (Carestream, Philips Healthcare, Netherlands). Imaging and demographic data were anonymized.

### Imaging interpretation

Two board-certified musculoskeletal (MSK) radiologists with 18 and 20 years of experience in MSK imaging independently reviewed all images and were blinded to clinical information and to each other’s scoring. Any disagreements were resolved by consulting a third radiologist with 22 years of experience in MSK imaging to decide on a final scoring. For US studies, both images and radiology reports were utilized in the review by the radiologists. For image-guided interventional procedures, reports and images were reviewed by the radiologists. All US studies and image-guided procedures in our institution were performed by one MSK radiologist.

Injuries were categorized according to the tissue types described in the International Olympic Committee (IOC) consensus statement on methods for recording and reporting of epidemiological data on injury and illness in sports [6, 7].

### Muscle and tendon

Muscle and tendon injuries were considered positive and classified as acute if at least one of the following findings was present: increased intramuscular or intratendinous hypersignal on magnetic resonance imaging (MRI) fluid-sensitive sequences representing edema, partial muscle or tendon fiber discontinuity, and complete muscle or tendon fiber discontinuity [8]. If edema was present, muscle injuries were then classified according to Peetrons (US) [9] or an MRI-adapted 3-scale [10] classification. According to Peetrons US classification, grade 1 indicates focal/diffuse bleeding with lesions < 5% of the muscle cross-sectional area, grade 2 indicates partial rupture with lesions from 5 to 50% of the muscle volume or cross-sectional area, and grade 3 indicates complete muscle rupture with retraction. According to the MRI-adapted 3-scale classification, grade 1 indicates injuries characterized by edema without architectural disruption, grade 2 indicates a partial tear with architectural disruption, and grade 3 indicates a complete muscle or tendon rupture. The presence of a fibrotic scar (well-defined distorted low signal intensity area on T1- and T2-weighted images on MRI; well-defined hyperechoic area on US) was also recorded and classified as chronic injury. Direct muscle injury (or muscle contusion) was defined as muscle edema not confined to myofascial or myotendinous junctions and crossing fascial planes [11]. Delayed-onset muscle soreness (DOMS) was defined as diffuse edema with preservation of muscle architecture and affecting the entire muscle belly or muscle group [8]. Tendinopathy was defined as tendon thickening with increased signal on fluid-sensitive sequences [12]. The absence of any of these findings was scored as imaging negative and not recorded as a muscle/tendon injury.

### Ligament

Ligament injury was considered positive if one of the following was present: (1) ligament thickening and high signal intensity on fluid-sensitive images (MRI) and hypoechoic (US) and (2) incomplete or complete ligament discontinuity [13, 14]. The distinction between acute or chronic ligament injuries was made by the following imaging signs: The ligament tears were classified as acute if they presented intrasubstance and/or surrounding soft tissue high signal intensity on fluid-sensitive sequences (edema) and chronic if no edema was present.

### Cartilage

Cartilage injuries were categorized as chronic degenerative or acute traumatic. Chronic cartilage injuries were represented by fibrillations, thinning, irregularities, and defects with obtuse margins. Acute cartilage lesions are represented by chondral defects with sharp angled margins. The International Cartilage Repair Society (ICRS) modified classification for MRI was applied for cartilage assessment [15]. Grade 0 indicated normal cartilage; grade 1 indicated regular chondral surface with an increased intrasubstance signal; grade 2 indicated erosions or fissures extending to less than 50% of the cartilage thickness; grade 3 indicated erosions or fissures extending to more than 50% of cartilage thickness; and grade 4 indicated cartilage defects extending to the subchondral bone. Cartilage injuries were accounted for each articular compartment.

Meniscal tear was considered positive if one of the following was present [16]: (1) intrameniscal high signal intensity linear area extending to the articular surface; (2) linear meniscocapsular junction high signal intensity on fluid-sensitive sequences; or (3) displaced meniscal fragments.

### Synovitis and capsulitis

Joint impingement is included in the synovitis/capsulitis pathology type according to IOC consensus statement. It is defined as joint capsule thickening, periarticular soft tissue mass or edema, marginal osteophytes, and bone marrow edema [17].

### Bone

Bone fracture was considered positive if a fracture line was identified on XR or CT images. The presence of callous formation indicated a chronic fracture. A fracture was considered positive on MR imaging if it showed bone marrow edema on T2-weighted images and a linear fracture line on T1-weighted images. Bone marrow edema was defined as high signal intensity within the bone marrow on fluid-sensitive sequences [12]. Bone contusion was defined as an ill-defined area of bone marrow edema in the knee and foot without fracture line and without underlying cartilage injury [12].

### Spine injuries

Vertebral spine injuries were additionally evaluated for the presence of vertebral body or posterior element fractures categorized within the category of bone injuries, and also evaluated for degenerative disc disease and disc herniation, according to the 2014 spine nomenclature consensus [18], which were categorized within cartilage injuries.

## Results

**Fig. 1:**
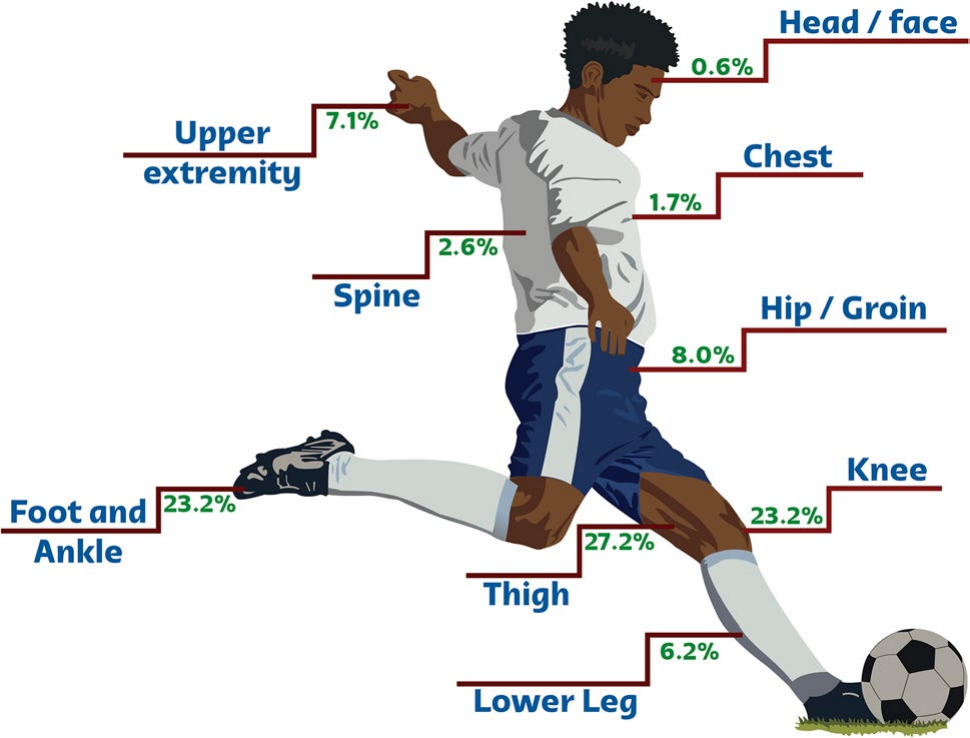
Frequency of imaging examinations for each body part

In total, 143 radiological examinations were performed for 94 players (mean age, 27 years ± 4 (SD); age range 19–39 years) from 28 national teams. Three national teams utilized other services in the centralized medical facility and did not request any imaging study. One team used a private healthcare facility for all medical services, including radiology. Considering the 31 national teams who utilized the polyclinic, we found an incidence of imaging utilization of 11.6% (94/806). Most injured players fell within the age range of 25 to 34 years old (71.2%, 67/94). The different types of imaging modalities and the concerned body parts are illustrated in Table 1, and the frequency of imaging examinations for each body part is shown in Fig. 1.

**Table 1:** Imaging modalities and correspondent body parts

Imaging modality	Ankle and foot	Lower leg	Knee	Thigh	Hip and groin	Upper extremity	Spine	Chest	Face	Total
MRI	15	8	18	38	11	3	3	0	0	96 (67)
XR	7	1	0	0	0	6	0	3	0	17 (12)
US	1	2	2	7	0	0	0	1	0	13 (9)
CT	2	0	0	0	0	1	0	2	1	6 (4)
CT-guided injection	0	0	0	0	0	0	1	0	0	1 (1)
US-guided injection	3	1	4	1	1	0	0	0	0	10 (7)
All	28	12	24	46	12	10	4	6	1	143

*MRI* magnetic resonance imaging; *XR*, radiography; *US*, ultrasound; *CT*, computed tomography. Data in parentheses are percentages

There were 17 players who underwent more than one imaging procedure for the same area and three who underwent more than one imaging procedure for different areas. All athletes who underwent an imaging-guided injection underwent a prior radiology procedure at our facility before injection.

Table 2 shows that most imaging examinations were performed for players from The Asian Football Confederation (AFC) and The Union of European Football Association (UEFA), with the AFC and The Confederation of African Football (CAF) having the highest utilization rates.

**Table 2:** Number of examinations by football confederations

	AFC	UEFA	CAF	CONMEBOL	CONCACAF
Teams	6	13	5	4	4
Players	156	338	130	104	104
Imaging studies	42 (29.3)	42 (29.3)	31 (21.6)	18 (12.5)	10 (7.0)
Utilization rate	(27)	(12)	(24)	(17)	(10)

Utilization rate = imaging studies/number of players. *AFC*, Asian Football Confederation;* UEFA*, Union of European Football Associations; *CAF*, Confederation of African Football; *CONMEBOL*, South American Football Confederation; *CONCACAF*, Confederation of North, Central America and Caribbean Association Football. Data in parentheses are percentages

In total, 112 injuries were reported for the 94 athletes who underwent an imaging examination. Table 3 shows the distribution and types of injuries.

**Table 3:** Number of imaging-detected injuries by type and anatomic location

Injury	Chest	Spine	Upper extremity	Hip and groin	Thigh	Knee	Lower leg	Ankle and foot	Total
Muscle and tendon	0	0	2	7	31	2	6	0	48 (42.8)
Ligament	0	0	1	0	0	10	0	17	28 (25)
Cartilage and synovitis	0	2	0	0	0	14	0	8	24 (21.4)
Bone	2	1	5	2	0	0	1	1	12 (10.7)
Total	2 (1.7)	3 (2.6)	8 (7.1)	9 (8.0)	31 (27.6)	26 (23.2)	7 (6.2)	26 (23.2)	112

Cartilage and synovitis includes meniscal, labral injuries and articular cartilage, osteochondral injuries, joint impingement, and vertebral spine disc hernia. Bone injury includes bone marrow edema and fracture. Data in parenthesis are percentages

A total of 21 imaging examinations (14%) were normal (no detected injuries), including 9 MRI (6%), 9 XR (6%), and 3 CT (2%). Most of the normal MRI were thigh examinations (*n* = 5) with clinical suspicion of a muscle tear. Most of the normal XR were foot examinations (*n* = 6) with clinical suspicion of a fracture.

### Muscle and tendon

**Fig. 2:**
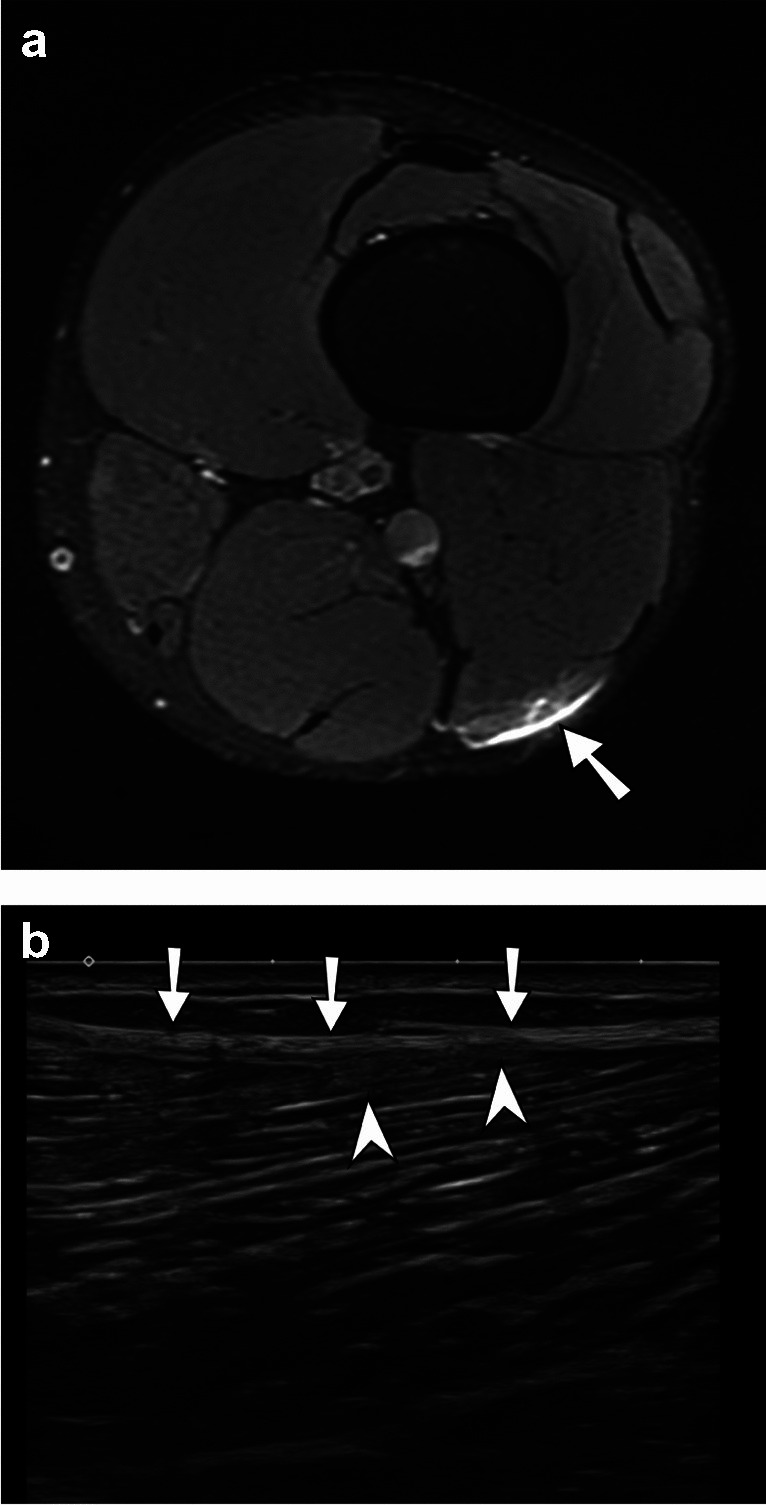
Muscle tear with MR/US correlation. **a** Axial T2-weighted fat suppressed MR image of the thigh. Myofascial tear with edema (arrow) at the posterolateral and distal aspect of the long head of the biceps femoris muscle, consistent with a grade 1 tear. **b** Corresponding longitudinal US image of the thigh shows aponeurotic thickening (arrows) with myofascial hyperechogenicity and loss of fibrillar pattern (arrowheads)

**Fig. 3:**
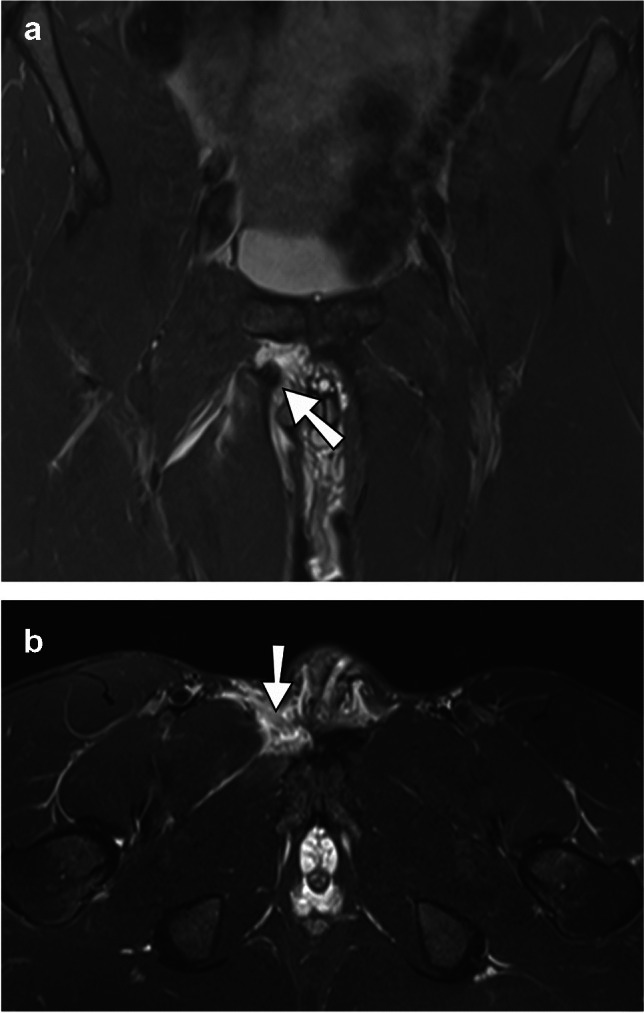
Complete tendon tear. Coronal (**a**) and axial (**b**) T2-weighted and fat-suppressed MR images of the groin region. Complete tear of the right proximal adductor longus tendon at the pubic ramus (arrow) with distal retraction, loss of muscle tension, and surrounding edema

A total of 46 muscle injuries in 42 athletes were recorded, and two athletes had injuries in two different muscle groups. Five athletes with clinical suspicion of muscle tear presented normal MRIs. The mean age of players with diagnosed muscle injuries on imaging studies was 27.6 years (SD 3.6 years), and the mean age of players with clinical suspicion of muscle injuries and normal MRIs was 23.8 years (SD 1.6 years), The observed age difference between the two groups is statistically significant (*p* < 0.05). In total, 54 MRI and nine US examinations were performed for the diagnosis of a muscle injury. MRI alone was utilized for the diagnosis of muscle injuries in 40 cases, US alone was used in three cases, and MRI combined with US was used in three cases. Eight athletes underwent more than one imaging examination in different dates, either to reassess a muscle injury or to evaluate a different muscle group. The vast majority of tears were found in the pelvis and lower extremities. Indirect muscle tears were reported in 40 examinations, accounting for 87% of all muscle injuries, with 21 (46%) grade 1 injuries (Fig. 2), 16 (35%) grade 2 injuries, and 3 (6.5%) grade 3 injuries (Fig. 3). Direct muscle injuries (or muscle contusions) were found in three examinations (6.5% of all muscle injuries) and DOMS was reported in 1 (2%) of cases. The vast majority of muscle tears were acute (95%–44/46) and there were two chronic muscle tears (4%–2/46).

Patellar and quadriceps tendinopathies were found in 2 (4.1%) cases of muscle and tendon injuries.

### Ligament

**Fig. 4:**
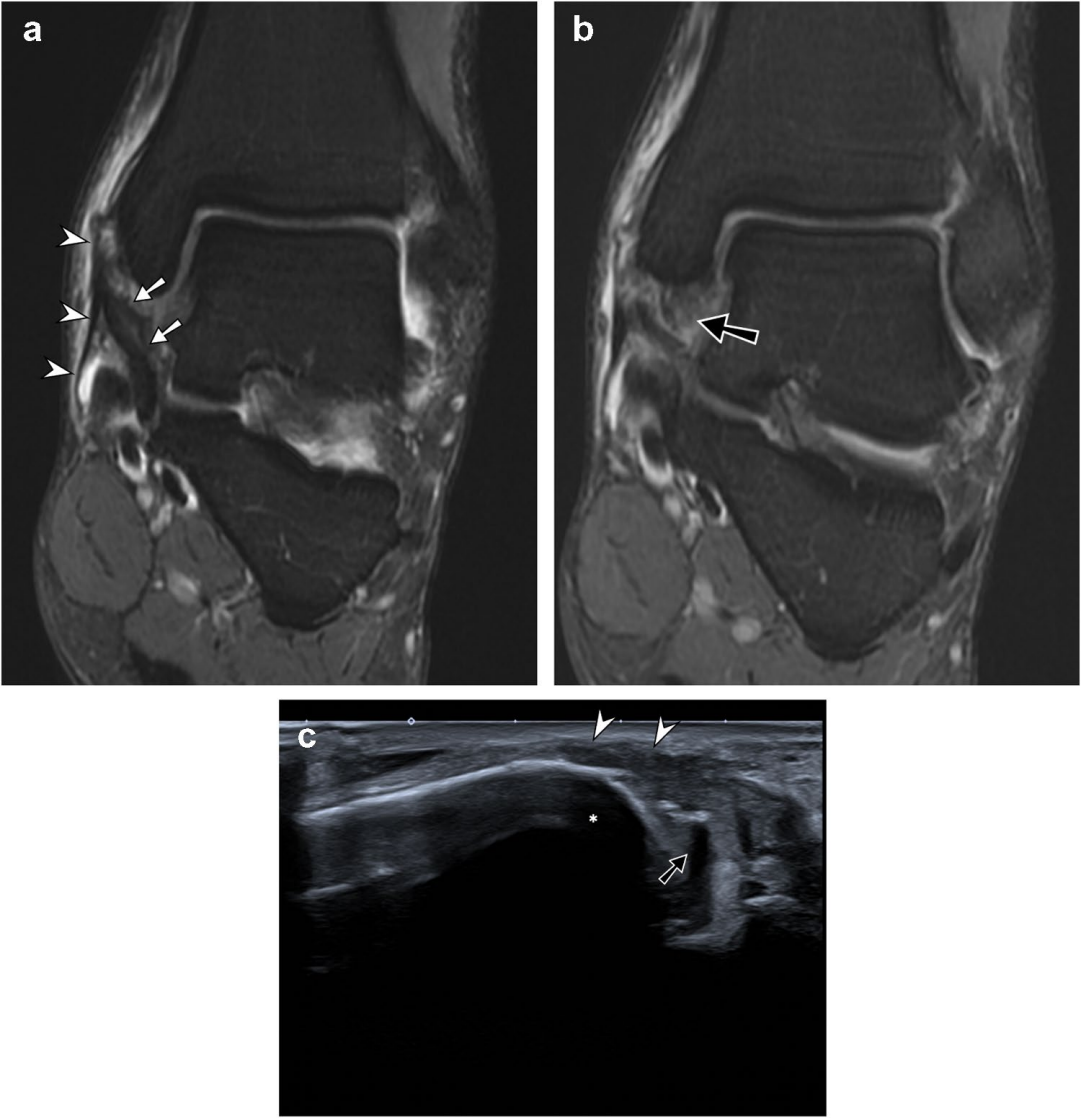
Ligamentous tear of the deltoid complex of the ankle—MR/US correlation. Coronal T2-weighted fat-suppressed MR images of the ankle show, in a more anterior image (**a**), tear of the superficial bundles of the deltoid ligament (white arrows) with flexor retinaculum tear (arrowheads) and periosteal stripping with edema. In a more posterior image (**b**), tear of the deep bundle of the deltoid ligament (black arrow) is demonstrated. Corresponding longitudinal US image (**c**) shows flexor retinaculum thickening and hypoechogenicity (arrowheads) at the medial malleolus (*) insertion with tear of the deep bundle of the deltoid ligament (black arrow)

Ligament tears were the second most common type of injuries (*n* = 28, 25%) and corresponded to the most common injuries in the knee and in the ankle regions. Anterior talofibular (ATFL) and calcaneofibular ligament (CFL) injuries were the most common ligament injuries (*n* = 12, 41%), followed by medial collateral ligament (MCL) injuries of the knee (*n* = 7, 24%). There were 9.5% (*n* = 2) of anterior cruciate ligament (ACL) injuries. Other injuries were deltoid ligament tears of the ankle (*n* = 3, 10.3%) (Fig. 4), ankle syndesmotic ligament tears (*n* = 1, 3.4%), tarsometatarsal ligament tears (*n* = 1, 3.4%), isolated low-grade posterolateral knee corner injuries affecting the arcuate and popliteofibular ligaments (*n* = 1, 3.4%), and an acute extrinsic ligament tear of the wrist (*n* = 1, 3.4%). All knee ligament tears were acute, and 77% (14/18) of the ankle ligament tears were acute. There was one previous ACL reconstruction surgery, intact.

### Synovitis and cartilage

Cartilage injuries were the second most common type of injuries in the knee and ankle and corresponded to 18% (20/112) of all injuries. The mean age of players with cartilage injuries was 28.3 years (SD ± 3.5). Most cartilage injuries of the knee were in the patellofemoral compartment, and all cartilage injuries of the ankle were at the talar dome. All cartilage lesions were classified as chronic degenerative.

Meniscal injuries accounted for 4% (5/112) of all injuries, including longitudinal, ramp, and isolated complex meniscal tears. Displaced meniscal fragment was found in one case. The medial meniscus was affected in 75% of all meniscal injuries.

Chronic anteromedial tibiotalar osteophytes, frequently associated with anteromedial impingement, were found in 1.7% (2/112) of all injuries.

Degenerative disc disease with disc herniations affecting the cervical and lumbar spines represented 1.7% (2/112) of all injuries.

### Bone

Acute bone fractures corresponded to 7.1% (8/112) of all injuries. Interestingly, most of the fractures occurred in the upper extremities and chest wall (87%, *n* = 7). Only one fracture affected the lower extremities.

Remaining bone injuries were metatarsal bone contusion (0.8%, *n* = 1), pubic bone marrow edema (1.7%, *n* = 2), and lumbar spine spondylolysis (0.8%, *n* = 1).

### Imaging-guided injections

Imaging-guided injections were performed on 11 players. Clinical indications were pain control in chronic chondral injuries of the knee, acute ankle ligament tears, acute muscle tears, and groin pain. There was one case of CT-guided epidural injection due to cervical spine disc extrusion. US-guided injections represented 91%, including four steroid injections for the knee (intraarticular treatment for pain related to cartilage injuries), two platelet-rich plasma (PRP) injections for the ankle (ligamentous injuries), two Traumeel injections for muscle injuries, and one steroid injection around the iliohypogastric nerve for a player with inguinal-related groin pain. Eight out of 11 players (72%) who undergone an image-guided injection were able to return to play during the tournament.

## Discussion

To the best of our knowledge, no data regarding imaging-detected musculoskeletal injuries or imaging-guided interventions in a major football event have been published. We present an overview of player imaging data for the first FIFA World Cup where most players attended the same centralized medical facility.

MRI was the most common imaging procedure during the 2022 FIFA World Cup accounting for 67% of all examinations performed. This is likely a result of the types of injuries, the high availability of the equipment, and the lack of cost limitations for the teams. All imaging examinations were free of charge for the teams and available at all hours during the tournament. Injury surveillance studies conducted during previous World Cups have reported that 79% of injuries were contusions, strains, and sprains [1, 2] which are the most likely to require MR investigations.

Imaging utilization rate at the central medical facility was 11.6%, reflecting a slight increase compared to the Rio 2016 Olympics. AFC teams had the highest utilization rate of imaging services (27%), closely followed by CAF (24%). For comparison, in the 2012 and 2016 Olympic Games, Africa had the highest percentage of athletes utilizing imaging services (15%), and Asia had one of the lowest utilization rates (4%) [3, 4]. The reason for this remains unclear. During the 2016 Olympics, the same trend was observed, leading to the hypothesis that the inadequate access to medical care in their home countries could be a contributing factor. However, the vast majority of players from African teams in the 2022 Soccer World Cup are employed by clubs outside Africa, particularly in Europe, which reduces the likelihood of limited access to medical care.

As expected, lower extremity injuries were the most common during the competition, which is in line with previous epidemiology studies of football injuries [19]. Upper extremity injuries were rare during the competition, accounting for only 7% of all radiological examinations and were most likely to be fractures, consistent with previous epidemiologic studies [20, 21].

Data from the Rio 2016 and Tokyo 2020 Olympics report a relatively low number of injuries in football players [5, 22]. Football is played by 16 teams in the men’s tournament at the Olympics, which is a number considerably lower the FIFA World Cup. Furthermore, in the men’s tournament, only three players over 23 years of age is allowed in the squad. Therefore, radiological observations in soccer injuries conducted at the last three summer Olympics are underestimated compared to the football World Cup and might be explained by some factors. First, in the Olympics, football players have a lower age range, and it was shown that aging might influence the responsivity of skeletal muscle to strain injury [23]. Second, there is a higher incidence of muscle injuries in tournaments with a higher perceived level of competition, such as the soccer World Cup [24].

Muscle injuries corresponded to 42% of all imaging-reported injuries at the central facility during the World Cup, thus making them the most frequent type of injury. This is comparable to the incidence of professional football muscle injuries found in a previous UEFA study (30–35%) [19, 25]. According to our data, the prevalence of muscle injuries in the World Cup was 5.2% (42 out of 806 athletes), which was much higher than the rates reported for soccer in the 2016 (0.6%) and 2020 (0.5%) Olympic Games [5, 22]. Considering the main differences between the World Cup and the Olympic tournament, this may be the reason for the increased rate observed in this study. For the first time, we have reported the classification of muscle injuries in a World Cup. According to the 3-scale classification, most of the muscle injuries were acute (95%) and grades 1 and 2 (80%), which was similar to what was reported for athletes at the 2016 Rio Olympics (85.2% of grade 1 and 2 muscle injuries) [22]. Additionally, it is worth noting that the group of players diagnosed with muscle injuries based on imaging studies had an older mean age compared to the group with clinical suspicion but normal MRIs. It has been demonstrated that age is related to the responsivity of skeletal muscle to strain injury [23], highlighting the potential impact of age as a factor in the likelihood of muscle injuries.

Despite the good accuracy of US in diagnosing and staging muscle injuries, MRI alone was utilized in 87% of all muscle injuries. US normally has some advantages over MRI: lower cost, wider availability, a higher spatial resolution, and the potential for dynamic evaluation. There is conflicting evidence in the literature regarding the clinical relevance and sensitivity of US compared to MRI. It has been shown that MRI is more sensitive for low-grade tears and soleus muscle injuries, and sensitivities are similar for hamstring tear evaluation [26–28]. The athletes competing in a football World Cup are among the best in the World and need a quick and accurate diagnosis, considering the short duration of the competition. As mentioned, cost and availability were not limiting factors for choosing the imaging method. Additionally, MRI, unlike US, is not operator-dependent and allows higher reproducibility in interpretation. We must also consider that it is a common practice for several national teams to have a portable US tool during the competition and even have a radiologist in the medical staff to perform US examinations, thus waiving the need for an US evaluation by external specialists.

The most common knee injuries were acute grade 1 and 2 MCL injuries, in accordance with previous studies [19, 29], which report MCL injuries as the second most common severe injury in football after hamstring muscle injury. In our data, MCL tears accounted for 6.4% of all injuries and were the third most common injury. It has also been reported to be the most common knee injury leading to time loss among football players [29].

During the World Cup, ATFL and CFL tears were the second most common injury and accounted for 44% of all ankle injuries. Deltoid and syndesmotic ligament injuries found during the World Cup are comparable with values found in another study (14% and 3% of all ankle sprains, respectively) [30]. The high prevalence of ankle cartilage injuries in our study is also in line with a previous study in asymptomatic football players (42% of all MRI examinations) [31] and should be taken in account when evaluating MRI scans.

Articular cartilage injury is a major concern for football players and is a major cause of disability and performance decrease in elite players. Progressive cartilage degeneration and osteoarthritis occur in up to 32% of the players and are proportional with the competitive level [32]. In the 2022 World Cup, chronic cartilage lesions were frequently found in the knee and ankle and represented 12% of all injuries. In the ankle, all cartilage injuries were found to be correlated with chronic ligament tears, while in the knee, approximately 40% of cartilage injuries were observed to be associated with a meniscal tear. These findings are in line with the existing literature, which indicates that osteoarthritis development in football players is unrelated to occurrence of macrotrauma [33].

In the pelvis and groin, adductor muscle and tendon injuries prevailed, aligning with previous studies that have shown adductor-related pain to be the most common type of groin pain, affecting 44–60% of athletes [34, 35].

Imaging-guided procedures were performed during the competition to help the athletes return to the playing field as quickly as possible. Musculotendinous, ligament, and cartilage injuries are the most common indications for guided injection therapies, which, despite conflicting evidence base in the literature, are becoming increasingly used among elite athletes. The goals are reduction of pain and accelerate tissue healing; the most common treatments are PRP, prolotherapy, hyaluronic acid, and steroid injections. Traumeel is a homeopathic preparation used to treat muscle injuries and has been shown to have similar effectiveness to non-steroidal anti-inflammatory drugs in enhancing recovery [36]. Indication for injections were based on the clinical approaches by the teams’ medical departments and discussion with radiologists. The majority of players (72%) who undergone an image-guided injection at the centralized medical facility were able to return to play during the competition.

This study had some limitations that need to be acknowledged: (1) scarce access to detailed clinical information, with lack of clinical and surgical correlation; (2) limited data on return-to-play times; (3) no access to frequency and details of US imaging performed by the participating member association medical teams; and (4) no access to imaging performed outside our institution.

In summary, 143 radiological examinations were performed in total, with MRI being the most frequent imaging modality (67%), the thigh the most frequent body area (32%), and acute grade 1 / 2 muscle injuries being the most frequent pathology type. Our study highlights the usefulness of imaging services during the 2022 FIFA World Cup providing epidemiologic data on radiology utilization, imaging-detected injuries, and imaging-guided injections.

## Data Availability

Data supporting this study cannot be made available due to confidentiality requirements by the event organizer.

## Abbreviations

ACL: Anterior cruciate ligament
AFC: Asian Football Confederation
ATFL: Anterior talofibular ligament
CAF: Confederation of African Football
CFL: Alcaneofibular ligament
CONCACAF: Confederation of North, Central America and Caribbean Association Football
CONMEBOL: South American Football Confederation
FIFA: Fédération Internationale de Football Association
IOC: International Olympic Committee
MCL: Medial collateral ligament
SD: Standard deviation
UEFA: Union of European Football Associations
